# 1-(Benzyl­ideneamino)pyridinum iodide

**DOI:** 10.1107/S1600536808043717

**Published:** 2009-01-08

**Authors:** Yong-Tao Cui, Jian-Qiang Wang, Chun-Xiang Ji, Cong-Ren Wu, Cheng Guo

**Affiliations:** a College of Science, Nanjing University of Technolgy, Xinmofan Road No. 5 Nanjing, Nanjing 210009, People’s Republic of China

## Abstract

In the title compound, C_12_H_11_N_2_^+^·I^−^, the aromatic rings are oriented at a dihedral angle of 73.40 (3)°. In the crystal structure, π–π contacts between the pyridine rings and the benzene and pyridine rings [centroid–centroid distances = 3.548 (3) and 4.211 (3) Å] may stabilize the structure.

## Related literature

For background, see: Okamoto *et al.* (1967). For bond-length data, see: Allen *et al.* (1987 ▶).
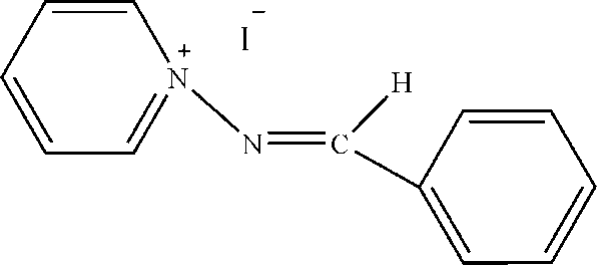


## Experimental

### 

#### Crystal data

C_12_H_11_N_2_^+^·I^−^
*M* *_r_* = 310.13
Monoclinic, 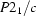

*a* = 10.5722 (17) Å
*b* = 7.8219 (13) Å
*c* = 15.386 (3) Å
β = 108.354 (2)°
*V* = 1207.6 (4) Å^3^
*Z* = 4
Mo *K*α radiation
μ = 2.62 mm^−1^
*T* = 291 (2) K
0.13 × 0.12 × 0.10 mm

#### Data collection

Enraf–Nonius CAD-4 diffractometer
Absorption correction: ψ scan (North *et al.*, 1968 ▶) *T*_min_ = 0.727, *T*_max_ = 0.780
5768 measured reflections
2133 independent reflections
1713 reflections with *I* > 2σ(*I*)
*R*_int_ = 0.067
3 standard reflections frequency: 120 min intensity decay: none

#### Refinement

*R*[*F* ^2^ > 2σ(*F* ^2^)] = 0.030
*wR*(*F* ^2^) = 0.075
*S* = 0.94
2133 reflections
136 parameters
H-atom parameters constrained
Δρ_max_ = 0.50 e Å^−3^
Δρ_min_ = −0.53 e Å^−3^

### 

Data collection: *CAD-4 Software* (Enraf–Nonius, 1989 ▶); cell refinement: *CAD-4 Software*; data reduction: *XCAD4* (Harms & Wocadlo, 1995 ▶); program(s) used to solve structure: *SHELXS97* (Sheldrick, 2008 ▶); program(s) used to refine structure: *SHELXL97* (Sheldrick, 2008 ▶); molecular graphics: *ORTEP-3 for Windows* (Farrugia, 1997 ▶); software used to prepare material for publication: *SHELXL97*.

## Supplementary Material

Crystal structure: contains datablocks global, I. DOI: 10.1107/S1600536808043717/hk2601sup1.cif

Structure factors: contains datablocks I. DOI: 10.1107/S1600536808043717/hk2601Isup2.hkl

Additional supplementary materials:  crystallographic information; 3D view; checkCIF report

## Figures and Tables

**Fig. 1.**
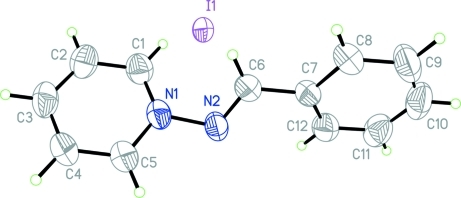
The molecular structure of the title molecule, with the atom-numbering scheme. Displacement ellipsoids are drawn at the 50% probability level.

## supplementary crystallographic information

### Comment

Some derivatives of 1-aminopyidium iodide are important chemical materials. We report herein the crystal structure of the title compound.

In the molecule of the title compound (Fig. 1), the bond lengths (Allen *et al.*,  1987) and angles are within normal ranges. Rings A (N1/C1-C5) and B (C7-C12) are, of course, planar, and they are oriented at a dihedral angle of 73.40 (3)°.

In the crystal structure, π-π contacts between the pyridine and the benzene rings and the pyridine rings, Cg1—Cg2^i^ and Cg1—Cg1^ii^ [symmetry codes: (i) x, 3/2 - y, z - 1/2; (ii) 1 - x, 2 - y, -z, where Cg1 and Cg2 are centroids of the rings A (N1/C1-C5) and B (C7-C12) , respectively] may stabilize the structure, with centroid-centroid distances of 3.548 (3) Å and 4.211 (3) Å.

### Experimental

For the preparation of the title compound, 1-aminopyridinium iodide (22.2 g, 0.10 mol) was dissolved in ethanol (20 ml), benzaldehyde(10.6 g, 0.10 mol) was added with stirring, and then the mixture was heated at reflux for 5 h. Upon cooling to room temperature, a precipitate formed, which was collected by filtration and washed with cold ethanol (2 X 10 ml) to obtain a yellow solid (yield; 21.7 g, 70%). Crystals suitable for X-ray analysis were obtained by slow evaporation of an ethanol solution.

### Refinement

H atoms were positioned geometrically, with C-H = 0.93 Å for aromatic and methine H and constrained to ride on their parent atoms, with U_iso_(H) = 1.2U_eq_(C).

### Crystal data

**Table:**

C_12_H_11_N_2_^+^·I^−^	*F*(000) = 600
*M**_r_* = 310.13	*D*_x_ = 1.706 Mg m^−^^3^
Monoclinic, *P*2_1_/*c*	Mo *K*α radiation, λ = 0.71073 Å
Hall symbol: -P 2ybc	Cell parameters from 25 reflections
*a* = 10.5722 (17) Å	θ = 2.1–25.3°
*b* = 7.8219 (13) Å	µ = 2.62 mm^−^^1^
*c* = 15.386 (3) Å	*T* = 291 K
β = 108.354 (2)°	Block, yellow
*V* = 1207.6 (4) Å^3^	0.13 × 0.12 × 0.10 mm
*Z* = 4	

### Data collection

**Table:**

Enraf–Nonius CAD-4 diffractometer	1713 reflections with *I* > 2σ(*I*)
Radiation source: fine-focus sealed tube	*R*_int_ = 0.067
graphite	θ_max_ = 25.0°, θ_min_ = 2.8°
ω/2θ scans	*h* = −12→12
Absorption correction: ψ scan (North *et al.*, 1968)	*k* = −9→7
*T*_min_ = 0.727, *T*_max_ = 0.780	*l* = −18→18
5768 measured reflections	3 standard reflections every 120 min
2133 independent reflections	intensity decay: none

### Refinement

**Table:**

Refinement on *F*^2^	Primary atom site location: structure-invariant direct methods
Least-squares matrix: full	Secondary atom site location: difference Fourier map
*R*[*F*^2^ > 2σ(*F*^2^)] = 0.030	Hydrogen site location: inferred from neighbouring sites
*wR*(*F*^2^) = 0.075	H-atom parameters constrained
*S* = 0.94	*w* = 1/[σ^2^(*F*_o_^2^) + (0.0365*P*)^2^] where *P* = (*F*_o_^2^ + 2*F*_c_^2^)/3
2133 reflections	(Δ/σ)_max_ = 0.001
136 parameters	Δρ_max_ = 0.50 e Å^−^^3^
0 restraints	Δρ_min_ = −0.52 e Å^−^^3^

### Special details

**Table:**

Geometry. All e.s.d.'s (except the e.s.d. in the dihedral angle between two l.s. planes) are estimated using the full covariance matrix. The cell e.s.d.'s are taken into account individually in the estimation of e.s.d.'s in distances, angles and torsion angles; correlations between e.s.d.'s in cell parameters are only used when they are defined by crystal symmetry. An approximate (isotropic) treatment of cell e.s.d.'s is used for estimating e.s.d.'s involving l.s. planes.
Refinement. Refinement of *F*^2^ against ALL reflections. The weighted *R*-factor *wR* and goodness of fit *S* are based on *F*^2^, conventional *R*-factors *R* are based on *F*, with *F* set to zero for negative *F*^2^. The threshold expression of *F*^2^ > σ(*F*^2^) is used only for calculating *R*-factors(gt) *etc*. and is not relevant to the choice of reflections for refinement. *R*-factors based on *F*^2^ are statistically about twice as large as those based on *F*, and *R*- factors based on ALL data will be even larger.

### Fractional atomic coordinates and isotropic or equivalent isotropic displacement parameters (Å^2^)

**Table:**

	*x*	*y*	*z*	*U*_iso_*/*U*_eq_	
I1	0.36542 (2)	0.37261 (3)	0.175113 (16)	0.05509 (13)	
N1	0.2501 (3)	0.8944 (3)	0.03899 (19)	0.0462 (7)	
N2	0.1744 (3)	0.8956 (3)	0.10105 (19)	0.0482 (7)	
C1	0.3602 (4)	0.7989 (5)	0.0544 (3)	0.0543 (9)	
H1	0.3942	0.7393	0.1091	0.065*	
C2	0.4222 (4)	0.7904 (5)	−0.0117 (3)	0.0597 (10)	
H2	0.4975	0.7225	−0.0024	0.072*	
C3	0.3734 (4)	0.8812 (5)	−0.0909 (3)	0.0586 (10)	
H3	0.4162	0.8775	−0.1353	0.070*	
C4	0.2612 (4)	0.9776 (6)	−0.1045 (2)	0.0633 (10)	
H4	0.2274	1.0402	−0.1582	0.076*	
C5	0.1982 (4)	0.9821 (5)	−0.0388 (2)	0.0617 (10)	
H5	0.1205	1.0454	−0.0484	0.074*	
C6	0.2447 (3)	0.9324 (4)	0.1829 (2)	0.0439 (8)	
H6	0.3347	0.9579	0.1961	0.053*	
C7	0.1831 (3)	0.9341 (5)	0.2553 (2)	0.0447 (8)	
C8	0.2456 (4)	1.0226 (5)	0.3354 (2)	0.0584 (9)	
H8	0.3250	1.0802	0.3419	0.070*	
C9	0.0654 (4)	0.8452 (5)	0.2465 (3)	0.0566 (10)	
H9	0.0240	0.7838	0.1934	0.068*	
C10	0.0110 (4)	0.8488 (5)	0.3166 (3)	0.0687 (12)	
H10	−0.0678	0.7902	0.3109	0.082*	
C11	0.0729 (5)	0.9391 (6)	0.3955 (3)	0.0748 (13)	
H11	0.0352	0.9419	0.4425	0.090*	
C12	0.1899 (5)	1.0252 (6)	0.4051 (3)	0.0716 (12)	
H12	0.2315	1.0851	0.4588	0.086*	

### Atomic displacement parameters (Å^2^)

**Table:**

	*U*^11^	*U*^22^	*U*^33^	*U*^12^	*U*^13^	*U*^23^
I1	0.05786 (18)	0.0622 (2)	0.04976 (17)	−0.00118 (11)	0.02344 (12)	0.00535 (11)
N1	0.0537 (16)	0.0507 (17)	0.0363 (15)	−0.0039 (13)	0.0172 (13)	−0.0040 (13)
N2	0.0509 (16)	0.0610 (18)	0.0355 (15)	−0.0047 (13)	0.0176 (13)	−0.0016 (14)
C1	0.064 (2)	0.050 (2)	0.052 (2)	0.0022 (18)	0.0222 (18)	0.0048 (18)
C2	0.066 (2)	0.055 (2)	0.067 (3)	0.0024 (19)	0.034 (2)	−0.005 (2)
C3	0.069 (2)	0.066 (2)	0.050 (2)	−0.012 (2)	0.0314 (19)	−0.013 (2)
C4	0.067 (2)	0.082 (3)	0.0402 (19)	0.001 (2)	0.0172 (18)	0.010 (2)
C5	0.061 (2)	0.083 (3)	0.043 (2)	0.010 (2)	0.0183 (17)	0.004 (2)
C6	0.0466 (18)	0.0441 (19)	0.0398 (19)	0.0008 (14)	0.0118 (15)	0.0004 (15)
C7	0.0471 (19)	0.0505 (19)	0.0351 (18)	0.0047 (15)	0.0107 (15)	0.0027 (16)
C8	0.061 (2)	0.070 (3)	0.0405 (19)	0.0007 (19)	0.0111 (17)	−0.0024 (19)
C9	0.054 (2)	0.068 (3)	0.050 (2)	−0.0020 (17)	0.0190 (18)	0.0039 (18)
C10	0.067 (3)	0.078 (3)	0.074 (3)	0.003 (2)	0.040 (2)	0.012 (2)
C11	0.097 (3)	0.082 (3)	0.062 (3)	0.033 (3)	0.049 (3)	0.019 (3)
C12	0.098 (3)	0.078 (3)	0.039 (2)	0.015 (3)	0.022 (2)	−0.004 (2)

### Geometric parameters (Å, °)

**Table:**

N1—N2	1.426 (4)	C6—C7	1.457 (4)
C1—N1	1.340 (4)	C6—H6	0.9300
C1—C2	1.375 (5)	C7—C8	1.385 (5)
C1—H1	0.9300	C7—C9	1.394 (5)
C2—C3	1.364 (5)	C8—C12	1.377 (5)
C2—H2	0.9300	C8—H8	0.9300
C3—C4	1.365 (5)	C9—C10	1.374 (6)
C3—H3	0.9300	C9—H9	0.9300
C4—C5	1.374 (5)	C10—C11	1.378 (7)
C4—H4	0.9300	C10—H10	0.9300
C5—N1	1.339 (4)	C11—C12	1.375 (6)
C5—H5	0.9300	C11—H11	0.9300
C6—N2	1.277 (4)	C12—H12	0.9300
			
C5—N1—C1	122.2 (3)	N2—C6—H6	120.2
C5—N1—N2	116.0 (3)	C7—C6—H6	120.2
C1—N1—N2	121.6 (3)	C8—C7—C9	119.8 (3)
C6—N2—N1	112.8 (3)	C8—C7—C6	118.8 (3)
N1—C1—C2	119.1 (4)	C9—C7—C6	121.4 (3)
N1—C1—H1	120.4	C12—C8—C7	120.0 (4)
C2—C1—H1	120.4	C12—C8—H8	120.0
C3—C2—C1	120.1 (4)	C7—C8—H8	120.0
C3—C2—H2	119.9	C10—C9—C7	119.7 (4)
C1—C2—H2	119.9	C10—C9—H9	120.1
C2—C3—C4	119.3 (4)	C7—C9—H9	120.1
C2—C3—H3	120.3	C9—C10—C11	120.1 (4)
C4—C3—H3	120.3	C9—C10—H10	120.0
C3—C4—C5	120.1 (4)	C11—C10—H10	120.0
C3—C4—H4	119.9	C12—C11—C10	120.5 (4)
C5—C4—H4	119.9	C12—C11—H11	119.7
N1—C5—C4	119.2 (4)	C10—C11—H11	119.7
N1—C5—H5	120.4	C11—C12—C8	119.9 (4)
C4—C5—H5	120.4	C11—C12—H12	120.0
N2—C6—C7	119.7 (3)	C8—C12—H12	120.0
